# Degradation Behavior and Lifetime Prediction of Polyurea Anti-Seepage Coating for Concrete Lining in Water Conveyance Tunnels

**DOI:** 10.3390/ma17081782

**Published:** 2024-04-12

**Authors:** Chengcheng Peng, Jie Ren, Yuan Wang

**Affiliations:** 1 College of Civil and Transportation Engineering, Hohai University, Nanjing 210024, China; 2 College of Water Conservancy and Hydropower Engineering, Hohai University, Nanjing 210024, China

**Keywords:** polyurea coatings, anti-seepage, accelerated aging test, cohesive strength

## Abstract

In the lining of water conveyance tunnels, the expansion joint is susceptible to leakage issues, significantly impacting the long-term safety of tunnel operations. Polyurea is a type of protective coating commonly used on concrete surfaces, offering multiple advantages such as resistance to seepage, erosion, and wear. Polyurea coatings are applied by spraying them onto the surfaces of concrete linings in water conveyance tunnels to seal the expansion joint. These coatings endure prolonged exposure to environmental elements such as water flow erosion, internal and external water pressure, and temperature variations. However, the mechanism of polyurea coating’s long-term leakage prevention failure in tunnel operations remains unclear. This study is a field investigation to assess the anti-seepage performance of polyurea coating in a water conveyance tunnel project located in Henan Province, China. The testing apparatus can replicate the anti-seepage conditions experienced in water conveyance tunnels. An indoor accelerated aging test plan was formulated to investigate the degradation regular pattern of the cohesive strength between polyurea coating and concrete substrates. This study specifically examines the combined impacts of temperature, water flow, and water pressure on the performance of cohesive strength. The cohesive strength serves as the metric for predicting the service lifetime based on laboratory aging test data. This analysis aims to evaluate the polyurea coating’s cohesive strength on the tunnel lining surface after five years of operation.

## 1. Introduction

Reinforced concrete has been widely used in the construction of civil infrastructure facilities. Concrete material is prone to cracking, and the water permeability of its material affects the long-term safety of the construction [1,2]. The development and utilization of sustainable composite construction materials play a crucial role in creating buildings that are not only environmentally friendly but also offer superior energy efficiency, leading to lower operational costs [3,4]. Currently, surface protective coatings are used to improve the impermeability of concrete. Research indicates that the effectiveness of these coatings is closely related to the elasticity, anti-seepage of the coating itself, and the appropriate bond strength between the substrates [5]. There are many kinds of surface coatings for concrete, including epoxy resin, polyurethane, polyurea, and acrylic acid. Even when using the same type of material, variations in formulations by different manufacturers can lead to differences in the final protective efficacy [6,7].

Polyurea elastomer is a high molecular polymer containing urea bonds (-NHCONH-) formed by the reaction of isocyanate (A component) and amine compounds (R component). Polyurea is a block copolymer composed of alternating soft and hard segments [8]. Polyurea’s excellent mechanical properties can be attributed to the presence of physical crosslinking. This crosslinking arises from intermolecular and intramolecular bidentate hydrogen bonds formed between the urea linkages [9]. Polyurea is a versatile material with a wide range of applications in construction and other industries [10].

As a new type of polymer material, polyurea exhibits excellent wear and corrosion resistance [11]. It also demonstrates strong bonding capabilities with various substrates such as steel, wood, and concrete, making it an outstanding choice for surface protective coatings [12,13,14]. After completing the pouring and maintenance of a concrete construction, applying a spray coating to its surface can enhance the durability of the structure. Therefore, polyurea can serve as an effective surface sealing material for expansion joints in concrete linings within water conveyance tunnels. It offers advantages such as ease of construction, anti-seepage, and adaptability to expansion joint deformations. However, practical engineering applications may encounter challenges, leading to polyurea coating failure. Research indicates that coating deterioration is often linked to environmental factors such as ultraviolet exposure, temperature fluctuations, air and water infiltration, and so on. Numerous scholars have studied the degradation mechanisms of concrete surface coatings in different environments [15,16,17,18,19,20]. The hydrothermal aging of polyurea materials is an irreversible chemical reaction between infiltrating water molecules and functional groups, leading to the cleavage of chemical bonds in polyurea elastomers [21]. High-energy ultraviolet radiation can cause the cleavage of active bonds in polymers, leading to an increase in the number of polar functional groups [19]. Although several studies have shown that the thickness of polyurea coatings can vary depending on the ambient temperature [22], polyurea still exhibits excellent performance as a waterproofing coating. Polyurea coatings with thicknesses of 2 mm and 4 mm could meet the impermeability requirements of 2 mm and 5 mm cracks under the action of 300 m head water pressure [23]. The wear process of the polyurea elastomer protective material is stable, and the wear loss is linear with the time of abrasion [24]. In summary, polyurea materials exhibit excellent mechanical properties, making them well-suited for the long-term operational environment of water conveyance tunnels.

The adhesion of the coating is very important for the coating performance [25]. Horgnies et al. [26] employed a specialized peeling method and utilized scanning electron microscopy (SEM) and Fourier transform infrared spectroscopy (FTIR) to analyze the fracture energy between high-performance concrete and polyurea coating. They found that curing time, curing method, porosity of the concrete material, and surface roughness significantly influence the bond performance. Specifically, higher concrete surface roughness and porosity lead to improved bond performance. Garbacz et al. [27] used four distinct methods to characterize the roughness of concrete surfaces and examined the correlation between surface roughness and concrete bond strength. Their findings indicate that the surface roughness of concrete treated with steel shot blasting is higher than that treated with silicon sandblasting. Additionally, they noted that apart from surface roughness, the presence of cracks and loose concrete blocks are also critical factors influencing the bonding performance. Delucchi et al. [28] conducted tests on the crack-bridging ability and anti-seepage properties of four types of concrete coatings, including epoxy and polyurethane. They also proposed two experimental methods to assess coating permeability and one for evaluating coating bridging ability. The study concludes that appropriate coatings should be selected based on the specific environmental conditions of the concrete. Significant variations in the bond strength were observed before and after the water immersion test [29]. Additionally, Nguyen et al. [30] discovered that water can lead to the debonding of organic coatings from the metal substrate. They also developed a new technique for in situ measurement of water at the interface between the organic coating and substrate. In conclusion, several factors influence the bonding properties between polyurea and concrete, such as the concrete’s inherent strength, surface roughness, and the conditions at the bonding interface. Nevertheless, there remains a lack of research on the adhesion aging rule of polyurea material in the water conveyance tunnel.

In water conveyance tunnels, polyurea materials, as an anti-seepage coating on the lining surface, play an important role in protecting key parts such as structural joints. Interface damage and debonding between the coating and the substrate are the most common failure modes of coating protection. The key factor affecting the application of polyurea coating in water conveyance tunnels is to ensure good adhesion between the polyurea coating and the concrete lining. The pull-off bond test is one of the most common portable tensile test methods for measuring bond strengths between a coating and a concrete substrate in site [31]. To accurately predict the service life of polyurea materials and ensure the safe operation of tunnels, it is necessary to establish accelerated aging tests that can simulate real aging mechanisms effectively.

In this study, based on the analysis mentioned above and considering the environment of water conveyance tunnels, the primary environmental factors influencing the aging of polyurea coating are identified as water flow, water pressure, and temperature. Currently, there is a scarcity of studies and analyses focusing on these aging factors. Therefore, it is imperative to investigate the aging patterns of cohesive polyurea coatings in relation to the environment of the water conveyance tunnel. This research will enable the reasonable prediction of the service lifetime of polyurea coatings, thereby supporting the application of polyurea anti-seepage coating in tunnels.

## 2. Materials and Methods

### 2.1. Field Investigation (In Situ Testing)

**Figure 1:**
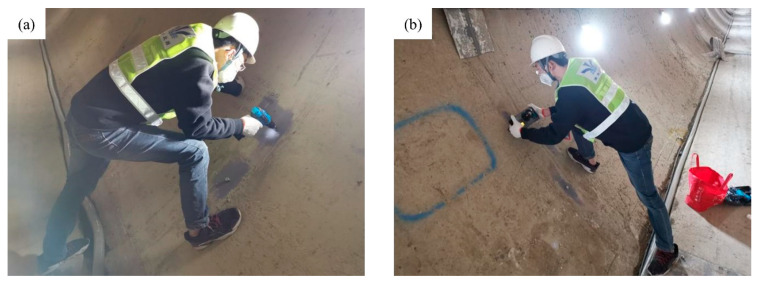
Pull-off test between polyurea coating and concrete lining substrates in the tunnel. (**a**) Cutting polyurea on site. (**b**) Pull-off test.

In the middle of November 2019, during the maintenance of the water supply in Zhengzhou, China, the tunnel, the polyurea material at the structural joint and anchor groove was investigated, and the bond strength between the polyurea material and the concrete lining of the tunnel was sampled and tested on site. The pull-off test is a dependable method that offers several benefits. It is a simple, reliable, and easy-to-use technique for evaluating the in situ strength of concrete and the bond strength between coatings and the concrete substrate in situ. Due to the smooth surface of polyurea, achieving a strong bond with the pull head for testing is challenging. This limitation hinders the use of adhesive failure tests to directly measure the bond strength between polyurea and the concrete matrix. During the test process, the sediment is first cleaned from the coating surface. The coating surface is then roughened using sandpaper. This roughening process ensures a strong bond between the coating and the pull head. After 24 h of adhesive curing of the pull head, the circumference of the pull head by the cutting device is used to penetrate the coating to the concrete matrix. Then, a cohesive strength measuring instrument (Proceq-Dy216) is used on the roughened surface to test the cohesive strength (Figure 1).

### 2.2. Accelerated Aging Test

#### 2.2.1. Preparation of Specimens

**Figure 2:**
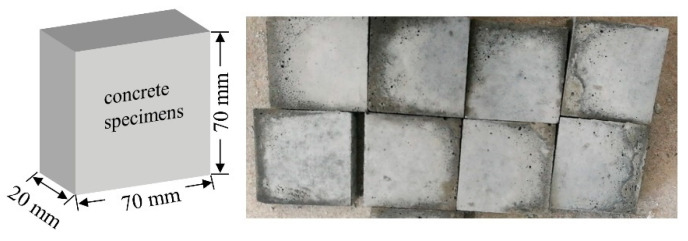
Diagram of concrete specimen preparation.

**Figure 3:**
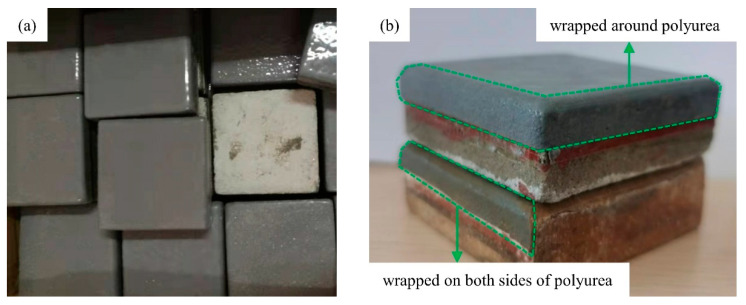
Concrete specimens after spraying polyurea elastomer. (**a**) The top surface of the concrete sample sprayed with polyurea coating. (**b**) The side of the concrete sample sprayed with polyurea coating.

The prism concrete specimens (Figure 2) were prepared with dimensions of 70 mm × 70 mm × 20 mm for the spraying polyurea coating, aiming to conduct the pull-off test. Commercial composite Portland cement P.O.42.5 was used in the specimens, with medium sand (fineness modulus between 2.3 and 3.0) and 5–10 mm diameter crushed stones mixed with pure water. The specimens underwent standard curing conditions (20 ± 2 °C, 95% relative humidity) for a duration of 28 days. The mix ratio of the concrete specimens was cement: water: sand: crushed stone: water reducer at 2.88:1:6.33:5.17:5.2%. Subsequently, the concrete surfaces were dried at 60 °C for 48 h until the moisture content dropped below 8%, following which the polyurea material was sprayed for pull-off testing (Figure 3).

The experiment used polyurea materials formulated by the China Institute of Water Resources and Hydropower Research in Beijing and produced primers (SKJ-001, SKJ-002) by Qingdao Ocean New Material Technology Co., Ltd. (Qingdao, China), as well as the Spray Polyurea Elastomer (SPUA)-SKJ II polyurea coating produced by the Joint R&D Production Base of Qingdao Jialian at the Marine Chemical Research Institute. In this study, the polyurea elastomer used for spraying was synthesized as A, B dual components. Component A comprises isocyanate, while component B contains amino polyether and terminal amino chain expansion agent, tailored specifically for applications in water conveyance tunnels in Zhengzhou, Henan Province. These additives enhance the material’s performance in low temperatures and humid environments and resist impact and wear. Two primers utilized were two-component silane-modified epoxy primer and polyurethane primer [32].

#### 2.2.2. Aging Test Device

**Figure 4:**
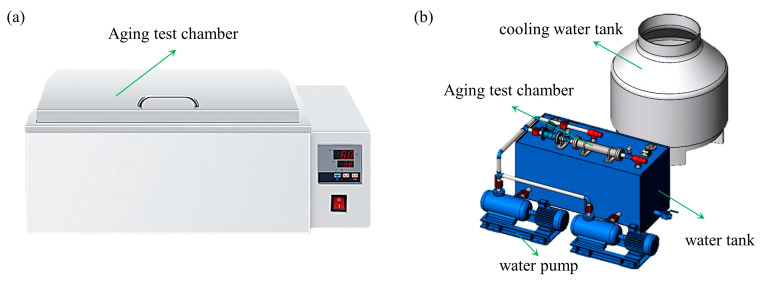
Schematic diagram of the test apparatus for the aging test. (**a**) Thermostatic water bath box. (**b**) Aging test equipment for simulating the operating environment of water conveyance tunnels.

Traditional accelerated aging experiments use hydrothermal aging methods. In this study, the first step is to conduct traditional hydrothermal aging experiments using a thermostatic water bath box with a heating temperature of 20–90 °C (Figure 4a).

The polyurea material in the water conveyance tunnel is mainly used for surface sealing and the anti-seepage effect of the joints in the lining structure. It is affected by multiple compound factors such as water flow impact, water pressure, and temperature changes in the tunnel for a long time. Therefore, this study combines the actual engineering environment and designs and manufactures an aging test device that can simulate the environmental conditions of the water conveyance tunnel site. It can be used to study the performance deterioration process of polyurea material under the combined effects of multiple factors such as temperature, water flow, and pressure in the tunnel (Figure 4b).

**Figure 5:**
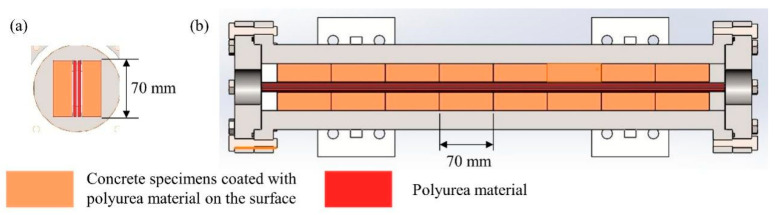
Schematic diagram of internal specimen placement of aging test chamber. (**a**) Left view of the aging test chamber. (**b**) Vertical view of the aging test chamber.

The apparatus replicates the conditions of a water conveyance tunnel, facilitating accelerated specimen aging through temperature elevation (Figure 4b). This device can examine the variation in cohesive strength of polyurea specimens under a composite influence of temperature, dynamic water flow, and pressure. The testing apparatus consists of a water tank, two constant water pumps, an aging test chamber, a cooling water tank, a temperature measurement, a flow meter, two pressure gauges, and a control device. To fulfill the demands of prolonged continuous operation, two constant water pumps operate alternately. A heating device and a temperature sensor were incorporated into the water tank to elevate the temperature and expedite specimen aging. Aligned with the real water flow conditions of a water conveyance tunnel in Henan Province, the test apparatus can simultaneously simulate aging environments with varying water pressures of up to 0.65 MPa (with a maximum water temperature reaching 80 °C). Concrete specimens coated with polyurea material on the surface, each measuring 70 mm by 70 mm by 20 mm, were positioned on both sides of the unit, with a polyurea material placed in the middle (Figure 5). During the experiment, water was heated to the designated temperature in a water tank. A water pump was used to continuously circulate water with a certain pressure and flow rate into the aging test chamber, simulating the operating conditions of a real water delivery tunnel.

#### 2.2.3. Experimental Design

In traditional accelerated aging experiments, a well-established approach to expedite the aging of polyurea coatings in the lab is to elevate the temperature. Align with the environmental conditions encountered within water conveyance tunnels, the combined effects of water and temperature aging factors were used to evaluate the cohesive strength variation. Concrete specimens coated with polyurea material were immersed in water at constant temperatures of 20 °C, 50 °C, 65 °C, and 80 °C. Subsequently, the degradation performance of cohesive strength was measured after 7-day and 21-day test cycles.

The test water temperatures were set at 50 °C, 65 °C, and 80 °C during indoor tests on the degradation behavior of polyurea materials in simulated on-site environments. The polyurea coating–concrete specimen was placed in the central slot of the primary testing apparatus to expose it to the combined effects of these factors. This configuration allows water to flow over the material’s surface, simulating a hydraulic tunnel’s internal water flow conditions. To prevent water erosion from undermining the bond between the polyurea and the concrete specimen, the polyurea coating was applied to the side of the specimen (Figure 3b). The aging period spans 1, 3, 7, 14, 21, 25, 27, and 28 days. Previous research indicates significant discrepancies in cohesive strength measurement results due to test instrument and method variations. Hence, to ensure comparability with field measurements, the cohesive strength of the specimen was also assessed using the same portable instrument (Prodeq-DY216) [33]. The following table (Table 1) details the various experimental designs employed for the aging test of polyurea coatings.

## 3. Results 

### 3.1. The Results of In Situ Testing

**Figure 6:**
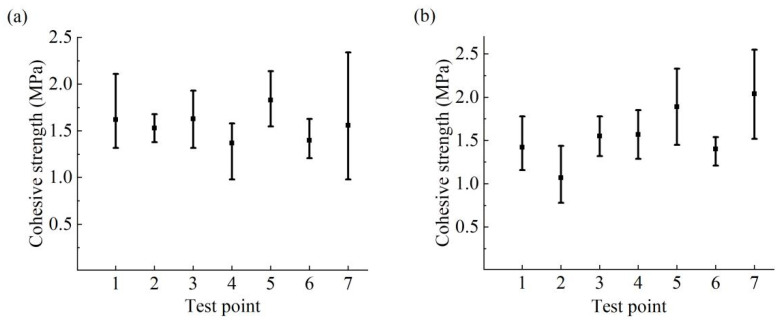
The cohesive strength of polyurea coating samples in Zhengzhou water conveyance tunnel. (**a**) Land sections of the water conveyance tunnel. (**b**) Underwater sections of the water conveyance tunnel.

In Zhengzhou Province, a water conveyance tunnel had been operating successfully for five years prior to the commencement of this testing. However, recent investigations have revealed the occurrence of bulging, rupturing, and water seepage in the polyurea material. The cohesive strength of polyurea coatings was evaluated at the land and underwater sections, with three repeated tests conducted near each sampling point. The following table (Table 2) details each sampling point. Test points 1 to 3 were chosen along the tunnel’s sidewall, points 4 to 6 on the tunnel floor, and point 7 at the anchor channel. The result of in situ testing is presented in the following figure (Figure 6). The average cohesive strength recorded for the tunnel was 1.827 MPa and 1.367 MPa, with minimum values of 2.035 MPa and 1.067 MPa, respectively. 

Notably, test results at the same position exhibited significant variability. It is noted that the cohesive strength between polyurea coating and the concrete lining can reach 2.2 MPa in wet or water environments. However, a substantial decrease in cohesive strength between polyurea and the concrete lining was observed along the tunnel’s length, compared to initial values, under the influence of the tunnel environment [32].

### 3.2. Aging Test Results in the Laboratory

#### 3.2.1. Hydrothermal Aging Test

In this section, the results of cohesive strength between polyurea coating and concrete after the hydrothermal aging tests are presented. The initial cohesive strength values for the three sets of samples between polyurea coating and concrete substrates tested in the laboratory were 3.829 MPa, 3.330 MPa, and 3.290 MPa, with an average cohesive strength of 3.493 MPa. The specimens coated with polyurea were immersed in water baths set at different temperatures, and changes in cohesive strength were recorded (Table 3). 

During the testing, the impact of temperature increase was considered. This led to water vapor accumulation between the polyurea and concrete surfaces, which resulted in accelerated bonding aging, which was evaluated using two specimens maintained at the same temperature (Figure 3b). As the test water temperature increased, the decline in cohesive strength became more pronounced. Notably, except for the 80 °C water temperature condition, the cohesive strength test results differed between the two specimens subjected to the same aging conditions. Specifically, when the test water temperature reached 80 °C, the cohesive strength of the cut specimen was significantly higher than that of the uncut specimen during the same test period. This analysis suggests that the uncut specimen is more prone to water vapor accumulation at the bonding interface between polyurea and concrete under high water temperature conditions, exacerbating the aging of the bonding material.

#### 3.2.2. Aging Test under Simulated On-Site Environment

**Figure 7:**
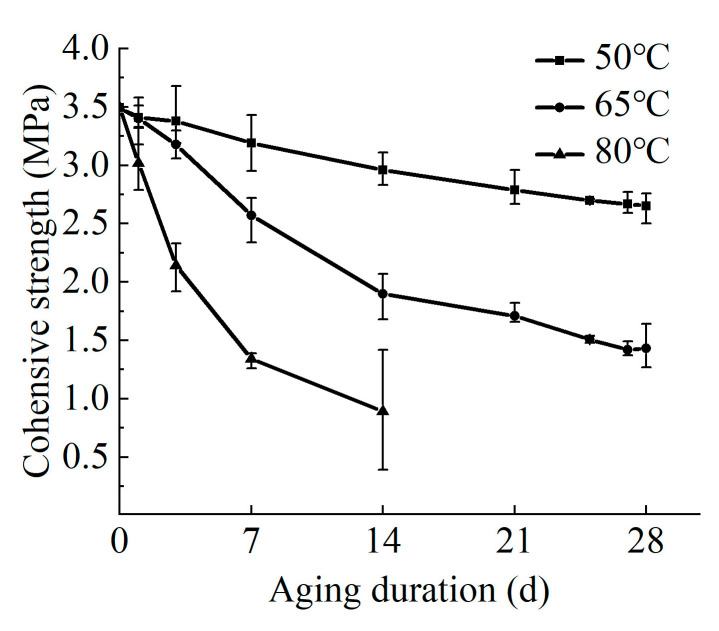
The variation of cohesive strength after simulated water tunnel environmental aging test over time at different temperatures.

This section presents the experimental results of aging tests under a simulated on-site environment. The effect of aging on cohesive strength is shown in the following figure (Figure 7). 

As expected, increasing ambient temperature and test duration significantly reduced cohesive strength. This effect was particularly pronounced at 80 °C, where a rapid decline in cohesive strength was observed, potentially leading to debonding after a short test period. These findings are consistent with previous results of the hydrothermal aging test. Notably, comparative analysis revealed that cohesive strength under water flow and pressure conditions was superior to that observed in the current test, highlighting the influence of these factors.

## 4. Discussion

### 4.1. Failure Types Analysis

**Figure 8:**
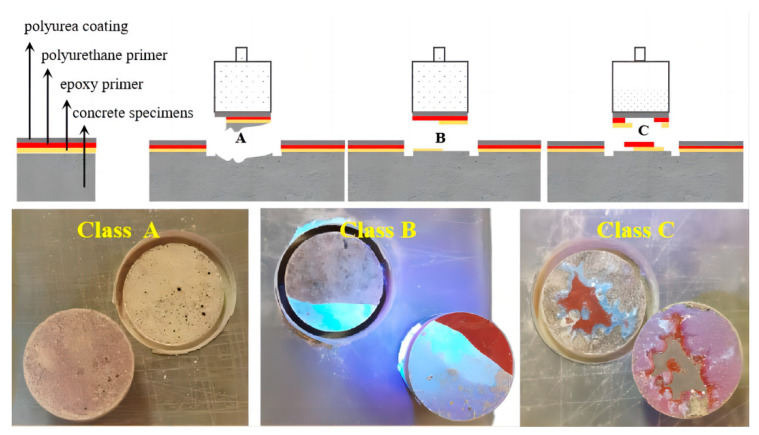
Specimens of the three different bond failure types.

To aid in understanding the failure mechanisms, Figure 8 defines and summarizes the various failure types observed during the aging tests. Based on the different bond failure types observed after cohesive strength measurement, the specimens can be classified into three categories: A, B, and C. Type A damage can be summarized as cohesive failure between concrete and epoxy resin, type B damage can be summarized as interfacial failure between the epoxy resin and polyurethane primer, and type C damage can be summarized as interfacial failure between polyurea and primer.

#### 4.1.1. Failure Types of the Hydrothermal Aging Test

**Figure 9:**
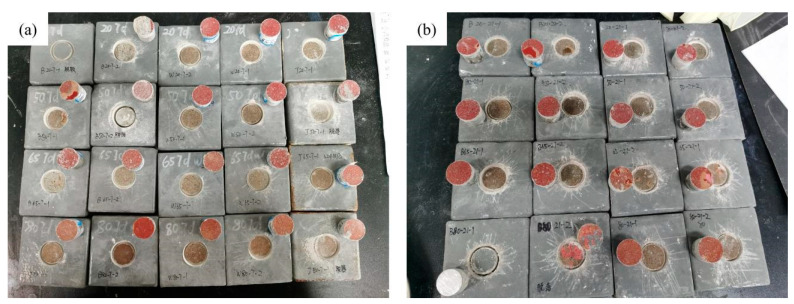
The failure types of polyurea–concrete specimens after the hydrothermal aging test. (**a**) Aging duration is 7 days. (**b**) Aging duration is 21 days.

This section illustrates the bond failure types of polyurea–concrete specimens after the hydrothermal aging test (Figure 9). The first row in the figure shows the specimens at a water temperature of 20 °C, the second row shows the specimens at 50 °C, the third row shows the specimens at 65 °C, and the fourth row shows the specimens at 80 °C. Under the experimental aging duration of 7 days, the failure modes of almost all specimens were of class A. Under the aging duration of 21 days, when the test water temperature was 65 and 80 °C, the failure types of the test specimens appeared to be of class B/C. 

Under mild aging conditions (short duration, low temperature), class A failure was observed, characterized by an adhesive failure within the concrete layer and residual concrete adhering to the epoxy surface. As aging time increased, debonding at the concrete–epoxy interface became the dominant failure mode. Notably, at 80 °C for 21 days, specimens wrapped around polyurea exhibited debonding with damage to the epoxy and polyurethane coatings. Red polyurethane primer degradation was particularly evident at 65 °C and 80 °C water temperatures, consistent with previous studies. Hydrothermal aging demonstrably weakens the adhesive performance of the epoxy layer. The observed failure progression suggests initial damage within the concrete substrates, debonding at the concrete–epoxy interface, and ultimately, with extended aging, degradation of the polyurethane primer and epoxy coating.

#### 4.1.2. Failure Types of the Simulated Tunnel Environment Aging Test

Following the simulated tunnel environment aging test, the bond failure types of the specimens were observed to be as follows. At a test water temperature of 50 °C, the specimens exhibited the failure type of class A. At a test water temperature of 65 °C, type B failure occurred after 21 days of the aging duration. At a test water temperature of 80 °C, type B/C failure occurred after only 7 days of the aging duration.

**Figure 10:**
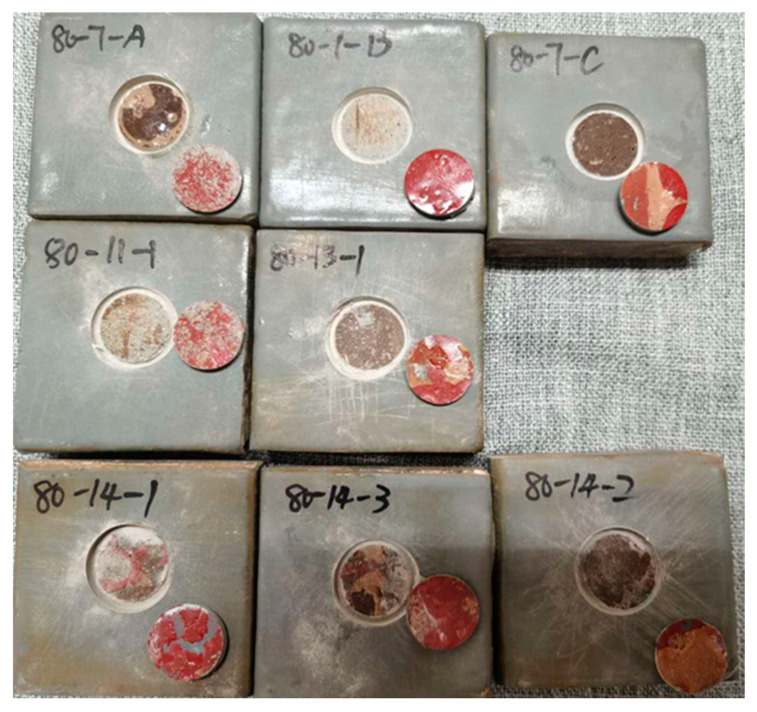
The bond failure types of specimens at 80 °C water during the aging test simulated in the tunnel environment.

Consistent with prior observations, at shorter aging times, failure is dominated by class A detachment, where the epoxy resin separates from the concrete base surface. As aging temperature and duration increase, the destruction mode transitions to class B and class C failures, characterized by the retention of epoxy and polyurethane undercoating on the exposed concrete surface. These findings corroborate those reported by other researchers [19,31,34]. At 80 °C water temperature, the adhesive interface degrades more rapidly due to water pressure (Figure 10). The first row in figure shows the bond failure types of specimens tested at 80 °C for 7 days. The second row shows the specimens tested for 11 and 13 days, and the third row shows the specimens tested for 14 days.We propose that water ingress along the concrete-polyurea interface triggers a reaction between the epoxy resin and polyurethane primer with water, leading to the observed bulges on some sample surfaces. By day 14, bond strength is significantly reduced, with an uneven polyurea–concrete interface exhibiting exposed gray polyurea coating in some areas.

#### 4.1.3. SEM Characterization of Typical Failure Types 

**Figure 11:**
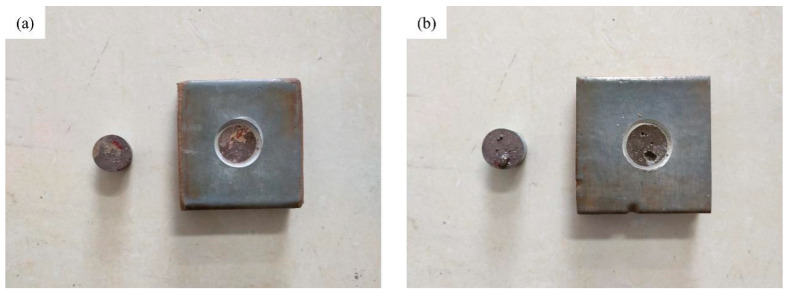
Macroscopic morphology of the fracture surface. (**a**) Case 3-8. (**b**) Case 3-1.

**Figure 12:**
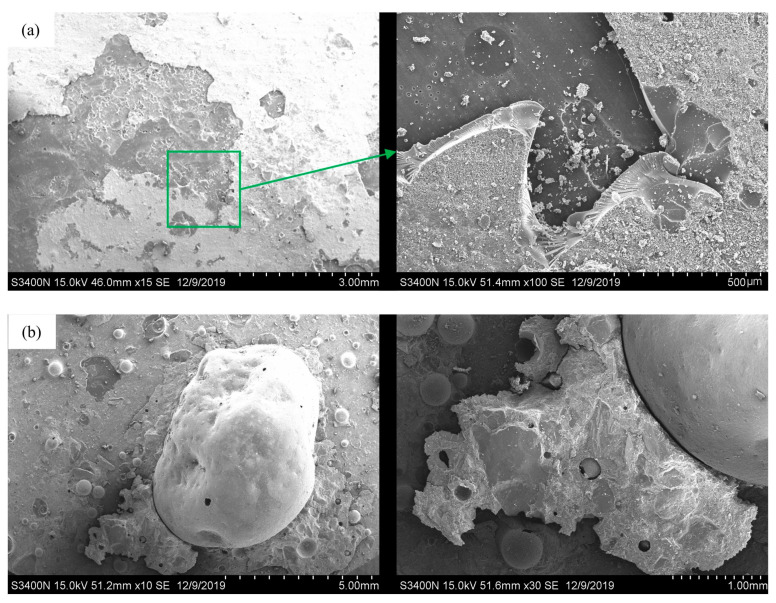
Micro morphology of the fracture surface. (**a**) Case 3-8. (**b**) Case 3-1.

Following pull-off strength testing, scanning electron microscopy (SEM) was employed to analyze the microscopic morphology of the fractured interface. The interfacial analysis after exposure to Case 3-8 is depicted in the following figures (Figure 11a and Figure 12a). Post-fracture analysis revealed the presence of residual epoxy resin and polyurethane undercoating on the concrete base surface. Microscopic magnification clearly visualized the coating and fracture texture (Figure 11b). A specimen from the test Case 3-1 observed that there were obvious defects on the concrete surface (Figure 11b). Preexisting defects necessitated epoxy resin repair before spraying. The fractured interface displayed visible epoxy resin and concrete, with the repair material completely filling the defect. High magnification revealed the epoxy resin–concrete interface with small adhering concrete fragments. The bond strength measured for this repaired specimen exhibited a slight increase compared to the standard test group under the same aging conditions. These findings suggest the potential utility of epoxy resin for mitigating concrete surface defects and enhancing the contact area, thereby improving the bond strength between the epoxy resin and the concrete surface.

### 4.2. Lifetime Prediction

This study employs artificial accelerated aging to simulate the effects of temperature, water pressure, and water flow on the cohesive strength and bond failure types between polyurea coating and concrete linings. Drawing on the test data in Section 3.2.2, this section predicts the polyurea’s service life for the project to guarantee operational safety. Kinetic equations, often expressed as exponential functions (Equation (1)), have been successfully employed to describe the relationship between aging performance (P) and aging time (t) for similar polymeric materials [35]. We will utilize this approach to predict the service life of the polyurea material.

(1) $$f\left(P\right)=e^{-Kt^{\alpha }}\;,$$

where *K* is the chemical reaction rate constant, unit min^−1^, *t* is the time, unit day, and *α* is the empirical constant. In terms of cohesive strength,$\;f\left(P_{\sigma }\right)=\frac{\sigma }{\sigma_{n}}$, σ is cohesive strength; $\sigma_{n}$ is the initial cohesive strength.

Differentiating Equation (1) results in Equation (2).

(2) $$\ln\left(P_{\sigma }\right)=A_{1}+B_{1}t^{\alpha }\;,$$

**Figure 13:**
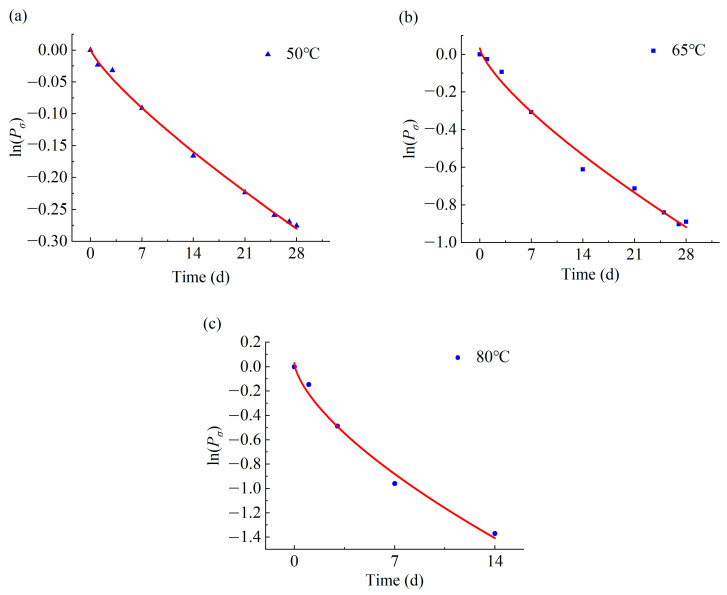
Relationship between cohesive strength and aging time. (**a**) Aging temperature is 50 °C. (**b**) Aging temperature is 65 °C. (**c**) Aging temperature is 80 °C.

Equation (2) was employed to establish a mathematical relationship between cohesive strength and aging time under varying temperature conditions within the simulated tunnel environment aging test. The following table (Table 4) presents the fitting parameters, while Figure 13 depicts the corresponding fitting curves.

Given the application of polyurea coatings in water conveyance tunnels, this study acknowledges the limitations of the traditional Arrhenius lifetime model, which primarily focuses on temperature. The Eyring reaction theory model incorporates nonthermal aging factors, and its expression is as follows.

(3) $$L\left(V\right)=\frac{1}{V}Const.\cdot e^{\frac{D}{V}}\;,$$

where *V* is the stress value in absolute units (such as relative humidity); *L(V)* is the life scale; *D* is the undetermined parameters of the model.

This study integrates the Arrhenius lifetime model with the Eyring reaction theory model to account for the combined effects of water and temperature on material aging, yielding a comprehensive water–thermal aging life model [36].

(4) $$L\left(H,T\right)=\frac{a_{1}}{H}e^{\frac{b_{1}}{H}+\frac{c_{1}}{T}}\;,$$

where *L*(*H*,*T*) is the water–thermal aging life model, *T* is the thermodynamic temperature, and *K*, *a*_1_, *b*_1_, *c*_1_ are the undetermined parameters of the model.

Following the cohesive strength results of the simulated tunnel environment aging test, when the humidity is considered, Equation (4) can be simplified to [37]:

(5) $$L\left(H_{0},T\right)=a_{H_{0}}e^{\frac{c_{1}}{T}}\;,$$

where $a_{H_{0}}=a_{1}e^{\frac{b_{1}}{H_{0}}/H_{0}};\;H_{0}$ is the constant relative to humidity, consistent with the Arrhenius model.

**Figure 14:**
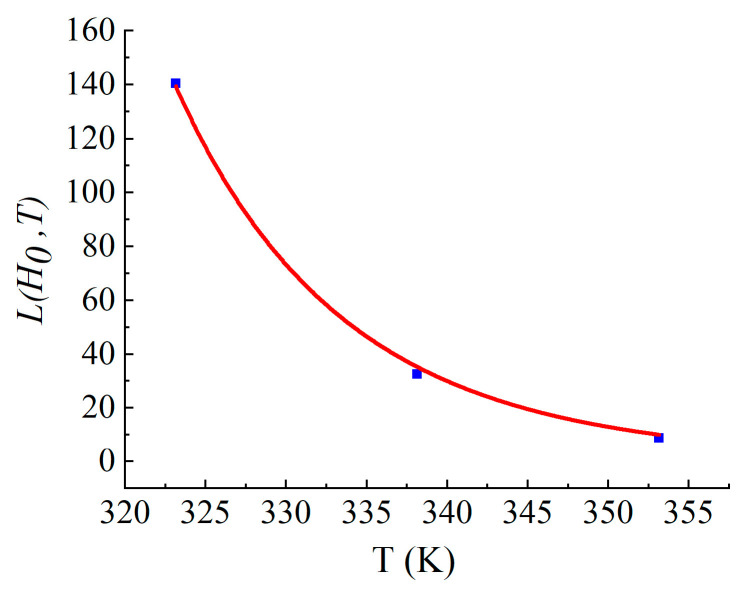
Fitting results of the water–thermal aging lifetime model.

According to the specification GB/T 23446-2009 [38], the bond strength of polyurea must exceed 2.5 MPa. The critical value of bond strength was set at 50% of this value. The critical value of cohesive strength was also set at 1.25 MPa. After fitting, the parameter substitution Equation (5) was obtained, as shown in Figure 14. If the annual average temperature of the water conveyance tunnel is 15 °C, the estimated service life of the polyurea coatings is approximately 16.52 years.

(6) $$L\left(H_{0},T\right)=4.96\times 10^{-12}e^{\frac{10008.5612}{T}}\;$$

**Figure 15:**
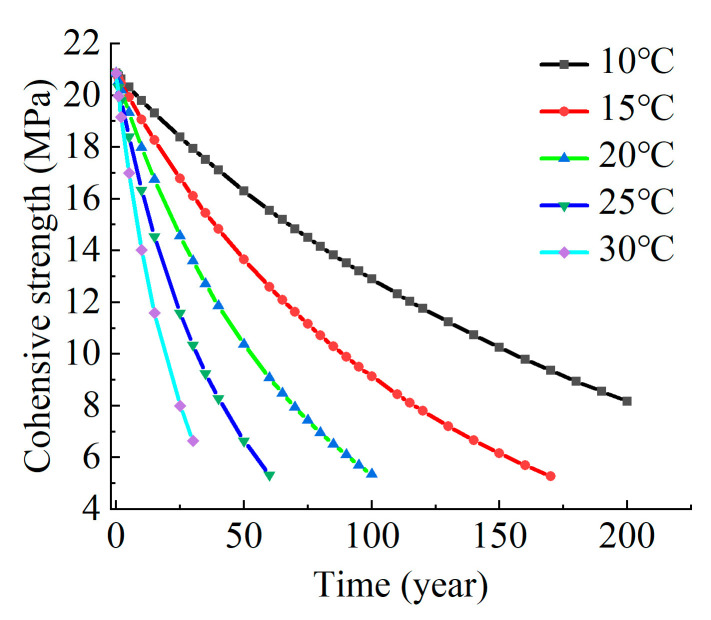
Predicted time-dependent cohesive strength profile.

The water–thermal aging lifetime model suggests a service life for the polyurea coating. This is attributed to the combined effects of temperature, water flow, and other factors encountered during real-world engineering applications, as reflected by the time-dependent cohesive strength profile in Figure 15. In the water conveyance tunnel project, with a predicted temperature range of 10~30 °C and a service life of 5 years, the measured cohesive strength of the polyurea exhibited a range of 0.83~2.93 MPa. The average estimated value was 1.56 MPa, with the lowest and highest values being 1.067 MPa and 2.035 MPa, respectively. These results demonstrate good agreement with the predicted values.

## 5. Conclusions

This study investigates the degradation of polyurea-sprayed coatings used in a water conveyance tunnel in Henan, China. The results reveal significant variations in the bond strength between the polyurea coatings and the concrete substrate after five years of operation, indicating environmental influence on bond performance.

An indoor accelerated aging test was designed to replicate the actual operating conditions of the water conveyance tunnel project, including temperature, dynamic water flow, and pressure. This test aimed to elucidate the deterioration of adhesive properties between the polyurea coatings and concrete lining. Three distinct failure modes were identified based on the observed debonding locations: (A) cohesive failure between concrete and epoxy resin, (B) interfacial failure between epoxy resin and polyurethane primer, and (C) interfacial failure between polyurea and primer.

The hydrothermal aging test demonstrated a progressive decrease in cohesive strength between the polyurea and the concrete substrates with extended aging time at a constant temperature. Notably, the aging test simulating the tunnel environment revealed that class A failure dominated at shorter aging times. However, with extended aging, the failure mode transitioned to class B failure and class C failure, characterized by debonding with residual epoxy and polyurethane primer on the exposed concrete surface.

The water–thermal aging lifetime model was employed to predict the service life of the polyurea coatings in the tunnel, with a threshold cohesive strength of 1.25 MPa, signifying acceptable bond performance. Assuming an annual average water tunnel temperature of 15 °C, this model successfully captured the aging trend of the polyurea–concrete cohesive strength, demonstrating good agreement with field investigations and testing.

This study designed an aging test device that simulates the real operating environment of water delivery tunnels. Indoor accelerated experiments were conducted to study the degradation law of the bond strength between polyurea and concrete under real conditions. The reasonable service life of the polyurea–concrete waterproof system in the tunnel environment was predicted. Considering the combined effects of temperature, water flow, and water pressure on the bonding performance of polyurea, a water–thermal life prediction model was constructed to predict its service life. In the future, more aging factors such as stress in the tunnel will be considered. The aging constitutive model of polyurea will be constructed, and the prediction model of its service life will be improved to predict its service life more reasonably.

## Figures and Tables

**Table 1:** Experimental design for aging test of polyurea coatings.

Experiment Type	Case	Aging Temperature (°C)	Aging Duration (d)
Hydrothermal aging test	Case 1-1	20	7
	Case 1-2	20	21
	Case 1-3	50	7
	Case 1-4	50	21
	Case 1-5	65	7
	Case 1-6	65	21
	Case 1-7	80	7
	Case 1-8	80	21
Aging test under simulated on-site environment	Case 2-1	50	1
	Case 2-2	50	3
	Case 2-3	50	7
	Case 2-4	50	14
	Case 2-5	50	21
	Case 2-6	50	25
	Case 2-7	50	27
	Case 2-8	50	28
	Case 3-1	65	1
	Case 3-2	65	3
	Case 3-3	65	7
	Case 3-4	65	14
	Case 3-5	65	21
	Case 3-6	65	25
	Case 3-7	65	27
	Case 3-8	65	28
	Case 4-1	80	1
	Case 4-2	80	3
	Case 4-3	80	7
	Case 4-4	80	14
	Case 4-5	80	21
	Case 4-6	80	25
	Case 4-7	80	27
	Case 4-8	80	28

**Table 2:** Sampling point of in situ testing.

Case	Testing Location	Chainage of Land Sections	Chainage of Underwater Sections
1	The wall of the structural joint	27-28	96–97
2		68–69	255–256
3		174–175	342–343
4	The bottom of the structural joint	27–28	96–97
5		67–68	255–256
6		174–175	342–343
7	Anchor channel	69	342

**Table 3:** Results of hydrothermal aging test.

Case	Wrapped around Polyurea				Wrapped on Both Sides of Polyurea			
	Values in MPa		Average	Standard Deviation	Values in MPa		Average	Standard Deviation
Case 1-1	*	3.72	3.72	-	3.05	3.82	3.44	0.39
Case 1-2	2.93	3.53	3.23	0.30	3.00	3.49	3.25	0.25
Case 1-3	3.28	-	3.28	-	3.84	3.38	3.61	0.23
Case 1-4	3.07	3.49	3.43	0.21	3.42	3.09	3.26	0.17
Case 1-5	2.52	2.97	2.75	0.23	3.10	2.77	2.94	0.17
Case 1-6	2.22	2.45	2.34	0.12	2.16	2.04	2.10	0.06
Case 1-7	1.63	1.50	1.57	0.06	2.18	1.92	2.05	0.13
Case 1-8	*	-	-	-	1.04	0.45	0.75	0.30

* Bonding adhesive failure at pull head.

**Table 4:** Fitting parameters at different temperatures.

Temperature/°C	Parameter			Correlation
	A1	B1	α	
50	0.00185 ± 0.00587	−0.01937 ± 0.00359	0.80354 ± 0.05232	0.99719
65	0.03319 ± 0.03828	−0.07949 ± 0.02575	0.74491 ± 0.09072	0.98999
80	0.03273 ± 0.08136	−0.25358 ± 0.07937	0.65825 ± 0.10981	0.98892

## Data Availability

The data presented in this research are available on request from the corresponding author.
